# The Disease of the Philistines

**Published:** 1901-05

**Authors:** 


### EDITORIAL NOTES AND COMMENTS.

The Business office of THE JOURNAL-RECORD is No. 64 Marietta Street.

The Editorial office is 318-319-320 The Prudential.

Address all Business communications to Dr. M. B. Hutchins, Mgr.

Make remittances payable to THE ATLANTA JOURNAL-RECORD OF MEDICINE.

On matters pertaining to the Editorial and Original Communications address Dr. Bernard Wolff, Atlanta.

Reprints of original articles will be furnished at cost price. Requests for the same should always be made *on the manuscript.*

We will present, post-paid, on request, to each contributor of an original article, twenty (20) marked copies of THE JOURNAL-RECORD containing such article.

### THE DISEASE OF THE PHILISTINES.

We read in the first book of Samuel how when the Philistines captured the ark and took it with them to their city of Ashdod and set it up in the temple of Dagon, the wrath of God was visited upon them in the destruction of their idol, a representation half human and half fish, and the appearance among them of a fatal disease called the emerods. All those smitten with the disorder died, and to appease the anger of the God of Israel, the ark was sent back on a newly made cart, to which were attached two milch kine. The cart contained in addition five golden images of the emerods and five golden mice according to the number of the Lords of the Philistines. It would be difficult to attribute to hemorrhoids the dreadful mortality attendant upon the disease described in the biblical narrative, for though harassing enough to fray the bonds of existence these pestiferous varicosities never wear them quite through. We are forced to cast about for another explanation of the fatality. According to Lichtenstein the disease of the Philistines was occasioned by the bite of a venomous spider, which attacked its victim when he was in the squatting posture obeying the calls of nature. The wound was received in the region of the anus or genitals. The spider, which is the solipuga, was as large as and somewhat resembled a mouse—hence the golden mice. Their bite causes tumefactions fatal in their consequence. He supposed that the solipugas were greatly multiplied among the Philistines by a special providence of God and caused the death of many individuals. Lichtenstein's explanation, while ingenious, lacks plausibility, and can hardly be sustained by contributory evidence from natural sources. It is scarcely possible that the bite of a spider could occasion such a mortality among a people or induce them to surrender a highly prized and dearly bought trophy of war as was the ark.

It is however worthy of note that in the tropics, as well as in some parts of the United States, there is a small species of arachnid, which has its habitat in privies, and sometimes bites those seated at stool. The injury is usually received in the anogenital region and is accompanied by brisk smarting and swelling, but is never fatal. Instances of this occurrence have been reported from California. The Texas tarantula, a large species of mygale, somewhat resembling the solipuga, is generally accounted venomous, and its bite is popularly regarded as fatal to the person attacked. Though universally dreaded, it is hardly conceivable that spiders could demoralize a nation.

Josephus adjudged the disease of the Philistines to be the dysentery from the word used to describe it, meaning in the Syriac the anus, and also the effort to evacuate the bowels. The fact that dysentery is very prevalent in the East and is often accompanied by a high mortality, lends some plausibility to the Josephine conception of the Philistine disease, but is by no means convincing.

An effort has been made to establish an identity between the disease of the Philistines and bubonic plague. Drs. Tidwell and

Dick published in the *Australasian Medical Gazette* an interesting article on the bubonic plague of 1141 B. C., which is reviewed in the *British Medical Journal.* They adduce their medical evidence from the scriptural account of the Philistine disease, from collateral sources and from philology.

The revised version gives the word tumor instead of emerods, and adds in a marginal note "plague boils as read by the Jews emerods." They quote Josephus, who refers to the Philistine endemic as a dysentery or flux, a very destructive disease. Those affected before death "brought up their entrails and vomited up what they had taken and what was entirely corrupted by the disease."

It would appear from the medical evidence that there was a severe epidemic of an infectious disease among the Philistines. The disease was sudden and violent in its onset. The symptoms consisted of emerods in the secret parts, vomiting of foul matter and dysentery.

As to the significance of these words the emerods is taken to mean hemorrhoids or piles, but this is a matter of dispute. The same word is used to designate a circumscribed swelling or a tumor, and the secret parts refer to the groins as well as the anal region. The vomiting of corrupt matters probably refers to hemorrhage from the stomach—the black vomit. Dysentery indicates a passage of blood from the bowels. Hemorrhage took place, therefore, from the mouth and from the rectum, as is usual in the plague. With these signs and symptoms given, the authors are satisfied the clinical picture of pestis minor is complete. The reference to mice would represent either a symbol of destructiveness, or carried the inference that they "marred the land," not from destructiveness, but by their dead and putrefying bodies, having been killed in great numbers.

The association of great fatality among rats and mice with the occurrence of plague is well known, and is taken by the authors as an additional prop to their theory of the identity of the disease of the Philistines with the bubonic plague. The date ascribed is 1141 B. C., or about the time at which the disease was described in 1 Samuel, which is at least 800 years before the first accepted appearance of the plague. A correspondent of the *British Medical Journal* calls attention to a passage in the 78 Psalm which refers to the events recorded in 1 Samuel. "He smote his enemies in the hinder parts, and put them to a perpetual reproach," conveys the idea that the emerods were an anal rather than an inguinal affection. To increase their disgrace and degrade them the more the affection was visited upon the obscene portions of the body. The question cannot be settled definitely, but the above seems to be an approximate solution gained by assembling circumstantial evidence and placing it more or less in agreement with our modern conception of a scourge which we have no reason to believe is not of great antiquity.

It is on the contrary perfectly possible to believe from precedent in the biblical narrative that an ordinarily trivial affection may have been miraculously aggravated to work out a divine purpose.

